# Effects of Zoledronate on Mortality and Morbidity after Surgical Treatment of Hip Fractures

**DOI:** 10.1155/2016/3703482

**Published:** 2016-03-22

**Authors:** Ömer Cengiz, Gökhan Polat, Gökhan Karademir, Oytun Derya Tunç, Mehmet Erdil, İbrahim Tuncay, Cengiz Şen

**Affiliations:** ^1^Department of Orthopedics and Traumatology, Faculty of Medicine, Bezmialem University, Fatih, 34050 Istanbul, Turkey; ^2^Department of Orthopedics and Traumatology, Istanbul Faculty of Medicine, Istanbul University, Çapa Fatih, 34050 Istanbul, Turkey; ^3^Muş State Hospital, Department of Orthopedics and Traumatology, 49100 Muş, Turkey; ^4^Department of Orthopedics and Traumatology, Istanbul Medipol University, Bağcılar, 34214 Istanbul, Turkey

## Abstract

We aimed to evaluate the effects of intertrochanteric femoral fractures on mortality, morbidity, and cost of zoledronate treatment in elderly patients treated by osteosynthesis. Based on Evans classification, 114 patients with unstable intertrochanteric femoral fractures were treated with osteosynthesis. After the surgical treatment of intertrochanteric fractures, the treatment group (M/F, 24/32; mean age, 76.7 ± SD years) received zoledronate infusion, and the control group (M/F, 20/38; mean age, 80.2 ± SD years) received placebo. Postoperative control visits were performed at 6-week, 3-month, 6-month, and 12-month time points. Functional level of patients was evaluated by the modified Harris hip score and Merle d'Aubigné hip score. By 12 months, the mean HHS in treatment and control groups was 81.93 and 72.9, respectively. For time of death of the patients, mortality was found to be 57.1% (16/28) on the first 3 months and 92.9% (26/28) on the first six months. The mortality rate in the treatment and control groups was 14.3% (8/56) and 34.5% (20/58), respectively. The use of zoledronic acid after surgical treatment of intertrochanteric femoral fractures in osteoporotic elderly patients is a safe treatment modality which helps to reduce mortality, improves functional outcomes, and has less side effects with single dose use per year.

## 1. Introduction

Intertrochanteric femoral fractures are often seen in the 6th and 8th decades and associated with morbidity, decrease in daily functions, and mortality in elderly patients [1, 2]. Although mortality rates range from 15% to 30%, the femoral fractures particularly show an increase within the year after the fracture occurs [2, 3]. As higher mortality and morbidity rates were reported with conservative treatment in intertrochanteric fractures, surgical treatment and early weight bearing are regarded as the standard approach [4]. Because of high rate of bone union in intertrochanteric fractures due to occurring in extra capsular and cancellous bone, rigid internal determination methods are considered as the first choice in surgical treatment [4, 5]. Osteoporosis has been considered as the main reason for 75% of the fractures in the elderly people. Nowadays, 1.3 million osteoporosis-related fractures were informed in the US annually, and 300,000 of these are hip fractures [6]. However, the data suggest that a small number of patients with hip fracture actually receive pharmacologic treatment for osteoporosis [7, 8]. Zoledronic acid is a potent bisphosphonate used in the treatment of osteoporosis. It has been especially used in the treatment of hip and vertebral fractures due to osteoporosis in postmenopausal women and has been shown in clinical studies to accelerate fracture healing and to inhibit the formation of new fractures [6, 7]. The purpose of this study is to assess the effects of zoledronic acid on mortality and morbidity of intertrochanteric hip fractures in the patients aged over 65 who underwent surgical osteosynthesis.

## 2. Materials and Methods

Hundred and twenty elderly (65 years or above) patients with intertrochanteric femoral fractures who underwent surgical osteosynthesis between the years 2012 and 2013 in our clinic were followed up for 1 year prospectively. The number of the patients was figured out by 95% confidence level and power analysis to decrease mortality rates from 25% to 15% and 55 patients were determined for each group.

Exclusion criteria were as follows: age below 65 years, pathological fractures, being bedridden prior to the fracture, any disease contraindicated to zoledronic acid, any disease that creates more than two comorbidities, trauma other than falling, and additional fractures to intertrochanteric femur fracture. The study started with 60 patients in each group. But 4 patients from the zoledronic acid group and 2 from the control group were excluded from the study due to the absence in control examinations. Thus, the study was completed with 114 patients.

All patients were informed about the procedure and were started to be treated after the approval. Patients were consecutively divided into 2 groups according to the sequential order of arrival and were randomized, and the first patient was included in the group to be given zoledronic acid (treatment group). All patients underwent surgical osteosynthesis and intertrochanteric antegrade nail (INTERTAN, Trademark of Smith & Nephew, Reg. US Pat. & TM Off.) of Smith & Nephew Company was selected as implant. All patients were asked to walk as much as they can tolerate with the help of a walker for postoperative 6 weeks.

All patients received 1200 mg of calcium carbonate and 1000 IU vitamin D for one year from postoperative day 1. In the treatment group, 5 mg of zoledronic acid was applied to each patient via 30 min IV slow infusion in postoperative week 2. After administration of zoledronic acid and two hours of observation, patients were discharged. Sodium chloride prepared and packaged in the same amount and size as 100 mL zoledronic acid bottles in our university's chemistry laboratory has been applied as placebo to the control group following the same procedure. All patients underwent bone mineral density measurements after postoperative day 1 and year 1. All patients were examined with radiological anteroposterior pelvis and two-way hip X-ray graph and functionally with the Harris hip score and the Merle d'Aubigné hip score at the 6th week, 3rd month, 6th month, and 12th month, postoperatively. In both groups, patients were categorized and examined according to the age, gender, affected side, the presence of additional disease, ASA (American Society of Anesthesiologists) score, timing of operation, the preferred type of anesthesia, postoperative complications, and mortality rates. Follow-up period was lasted for one year in both groups.

The study protocol was approved by local ethics committee (Date, 21 March 2013; number B.30.2.BAV.0.05.05 Bezmialem Foundation University, Medical Faculty Clinical Research Ethics Committee).

### 2.1. Statistical Analysis

A summary of the data was presented as mean, standard deviation, and percentage. Categorical data were analyzed by using chi-square test. Variations of functional scores in intragroup and intergroup combinations were compared with repeated ANOVA and post hoc Bonferroni method. Group mean survival times were estimated with Kaplan-Meier method. Comparison of mean survival times was investigated with Log-Rank test. Quantitative characteristics of the two groups were analyzed by *t*-test, for independent groups in terms of mean comparisons. Cox regression model was used to show which of multiple factors had significant effect on mortality. IBM-SPSS 20 program was used for analysis. In all tests, the level of significance was adjusted to 0.05.

## 3. Results

Among the 114 patients (M/F, 44 (38.6%)/70 (61.4)), 56 (49%) were treated with zoledronic acid (mean age, 76.79 years) and 58 (51%) with placebo (mean age, 80.28 years). In the treatment group, 24 (42.9%) patients were male and 32 (57.1%) were female. In the control group, 20 (34.5%) patients were male and 38 (65.5%) were female. Treatment and control groups were comparable in terms of gender distribution.

Patients were divided into two groups including the patients under 80 years old and over 80 years old and mortality rates were investigated. Mean age of 114 patients was 78.56 (56 patients in the treatment group with the average of 76.29; 58 patients in the control group with the average of 80.28). 92.8% of the patients who died (8/8 in the treatment groups; 18/20 in the control group) were found to be over 80 years old.

Patients were evaluated according to the ASA score at the preoperative stage and the patients with an ASA score of 5 were excluded from the study. 62 patients (54.4%) were evaluated as ASA 3-4 and 52 patients (45.6%) were evaluated as ASA 1-2. There was no statistical difference between the two groups with regard to the ASA values (*p* > 0.05). Considering the ASA and mortality relationship, mortality rate in the patients with ASA 3-4 was observed to be 78.4% (22/28).

The average operation time of the patients was observed to be 4 days (1–15). 76 patients (66.7%; treatment group/control group: 38/38) were determined as ≤4 days and 38 patients (33.3%; treatment group/control group: 18/20) were determined as >4 days and there was no significant difference between the two groups (*p* = 0.791).

While 32 patients (28.1%; treatment group/control group: 13/19) were operated on under general anesthesia, regional anesthesia was applied to 82 patients (71.9%; treatment group/control group: 43/39). There was no significant difference between the two groups in terms of type of anesthesia (*p* = 0.257).

The changes in bone mineral density of the surviving 86 patients at the end of postoperative 1st year were evaluated by DEXA in order to assess the efficacy of the treatment. Intergroup and intragroup differences were evaluated by *t*-test. The increase in bone mineral density in the treatment group was significant compared to the control group and was consistent with the literature (*p* < 0.01) (Table 3).

Twenty-eight (24.6%) of 114 patients, for whom average follow-up period was calculated as 9.5 months (treatment group/control group: 10.6/8.4), died at the end of postoperative 1st year. While the postoperative mortality rate in the treatment group was 14.3% (8/56), this percentage's being 34.5% (20/58) in the control group was remarkable. Considering the patients' time of death, the mortality rate in the first three months was observed 57.1% (16/28) and 92.9% (26/28) in the first 6 weeks. Then, the data were analyzed statistically between the two groups and statistically significant differences were found between the groups (*p* < 0.05) (Table 4).

Functional status of the patients was evaluated at the postoperative 6th week and 3rd, 6th, and 12th months by Harris hip score and Marlin d'Aubigné score. At the end of the postoperative 1st year, in the treatment group, average HHS was observed as 81.93/100 and average Marlin d'Aubigné score as 14.58/16, and in the control group, average HHS was observed as 72.9/100 and average Marlin d'Aubigné as 12.68/16. Significant difference was determined as a result of intergroup and intragroup statistical analysis (*p* < 0.05).

## 4. Discussion

Nine-tenth of hip fractures occur in people aged 65 and above. Half of these are intertrochanteric femoral fractures [4, 9]. High rate of incidence of intertrochanteric fractures has been found to be associated with advanced age, increased comorbidity, increased daily activity, and osteoporosis [10]. Particularly hip fractures associated with osteoporosis cause high rates of morbidity and mortality with costly long hospital stay and rehabilitation along with significant loss of function [11, 12]. Osteoporosis-related mortality may clearly be associated with death occurring within 6 months after the hip fracture [13]. In this study, the patients were divided into two groups to assess the effect of osteoporosis treatment on mortality and morbidity and some of them were treated pharmacologically. As the pharmacological agent, zoledronic acid, one of the bisphosphonates which is the current drug group of the osteoporosis treatment and administered as 30 min IV infusion once a year, was preferred.

Daily application of 5 mg IV zoledronic acid at the 90th day after the hip fracture has been determined to reduce the formation of new fractures by 35% and the risk of mortality by 28% [14]. In in vitro studies performed on mouse calvarium bone cultures, zoledronic acid was determined to inhibit vitamin D3 induced calcium release more potently compared to the other five bisphosphonates and to be 100 times more potent compared to pamidronate, 25 times compared to alendronate, 200 times compared to clodronate, 10 times compared to ibandronate, and 5 times compared to risedronate [15].

However, the use of oral bisphosphonates with stringent and complex procurement requirements is becoming very difficult for patients taking a large number of oral medications because of the damage in cognitive and functional status. As a result, poor adherence to the treatment with oral bisphosphonates leads to incomplete treatment [16, 17].

In the study about the timing of zoledronic acid, Eriksen et al. have shown that the medicine reached the maximum concentration at the postoperative 2nd week and had the highest efficiency in reducing mortality [18]. We also applied zoledronic acid to our patients at postoperative 2nd week.

Another point is the calcium and vitamin D support. Increased bone metabolism requires high amounts of calcium and vitamin D for fracture healing and remodeling. Although there are studies suggesting that oral calcium supplementation reduces the incidence of fractures, it can be considered as adjunctive therapy in the treatment of osteoporosis [19, 20]. We have given to all patients from the postoperative first day oral medication containing 1200 mg/day of calcium and 1000 IU of vitamin D.

Patients in both groups were categorized and assessed according to the age, gender, presence of comorbidities, the type of fracture, ASA (American Society of Anesthesiologists) score, osteoporosis degree, the time for surgery, preferred type of anesthesia, and the surgery performed. Although there was no significant difference between the two groups in terms of gender, ASA, time of operation, and type of anesthesia, there was statistical difference between the two groups in terms of age and type of fracture (*p* < 0.05).

Considering the literature, intertrochanteric fractures appear in women two times more than men [21, 22]. In our study, the distribution was 70 (61.4%) females and 44 (38.6%) men. There was no difference between groups in terms of gender distribution. Similar results have been observed in women (18/70; 25.7%) and males (10/44; 22.7%) related to the mortality rates.

The effect of surgical time on patient mortality still continues to be a topic of discussion. Generally accepted approach is to correct the medical condition of the patient immediately and then undergoing surgical procedure [4, 9]. In a retrospective study that included patients over 60 years old, the time until surgery in patients whose medical conditions have been improved has been proven not to have any effect on mortality and treatment of medical comorbidities has been concluded to be an advantage [23]. In our study, the average time for the patients to be taken to surgery was determined 4 (1–15) days. 76 (66.7%) patients were operated on in 4 days or less, and 38 (33.3%) patients were operated on after 4 days. All patients underwent surgery after stabilization of the medical conditions. There was no significant difference between the two groups.

In a 10-year prospective study about hip fractures, mortality in patients with ASA 3-4 was found significantly higher [12]; however, in a retrospective review, mortality rates of the patients aged 65–84 with ASA 3-4 were found higher after hip fracture [24]. In our study, patients were evaluated according to the ASA classification and ASA 5 was excluded. 62 (54.4%) patients were determined to be ASA 3-4 and 52 (45.6%) were ASA 1-2. Although there was no significant difference between ASA values between the groups, the effect of ASA on mortality has been evaluated and 22 (78.4%) of 28 patients who died were observed to be ASA 3-4.

Toker et al., in the study involving 1333 patients, related to the type of anesthesia in hip fractures, did not find any differences between the length of hospital stay of patients who received general and spinal anesthesia and the mortality rates [25]. In our study, regional anesthesia was administered to 82 (71.9%) patients, and general anesthesia was administered to 32 (28.1%) patients. No significant difference was determined between the two groups.

Risk factors associated with the occurrence of fractures become more widespread in increased ages; every 5 years of growth the risk of hip fracture increases 1.5–2 times [26]. The majority of intertrochanteric fractures occur over 70 years of age [4, 27, 28]. Lin et al. in the study involving 217 hip fractures have identified that the occurrence of trochanteric fracture and being over 80 years of age were a risk factor for mortality [29]. Wang et al., in a study evaluating 143595 patients with hip fractures retrospectively, have determined that mortality rates especially increased within the first year in patients over the age of 80 [30]. 114 patients over 65 years were enrolled in our study and the mean age was 78.56 (65–93) (Table 1). Patients in control group were found to be older (treatment group average age: 76.29; control group average age: 80.28) and were statistically significant. In addition, 92.8% of all patients who died (26/28) were determined to be 80 years and above. We believe that especially 80 years and above is a serious risk factor for mortality at the hip fractures.

Osteoporotic hip fractures are the most important causes of morbidity and mortality [31] and osteoporosis-related mortality may clearly be associated with deaths occurring within the 6 months after the hip fracture [32]. 5 mg of zoledronic acid with single dose IV annually is known to increase the bone mineral density within 1 year [14, 18, 33–35]. With the knowledge that DEXA measurements give more accurate results in the evaluation of bone mineral density [36, 37] and being the golden standard for osteoporosis detection [38], in our study, bone mineral density in the postoperative 1st year was assessed by DEXA. Intergroup and intragroup differences were evaluated by *t*-test. Zoledronic acid was determined to provide significant increase at the bone mineral density (Table 4) and was seen consistent with the literature [14, 39, 40].

**Figure 1:**
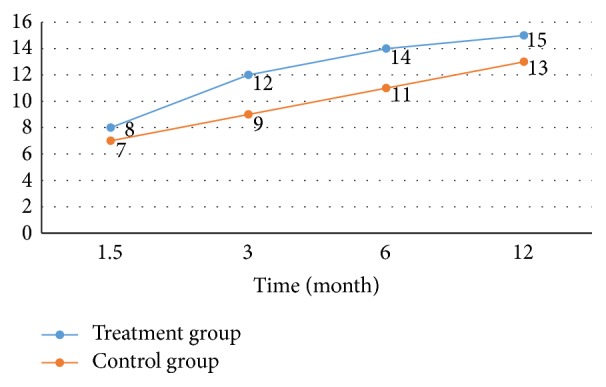
Intergroups changes on the Merle d'Aubigné scores for 12 months.

**Figure 2:**
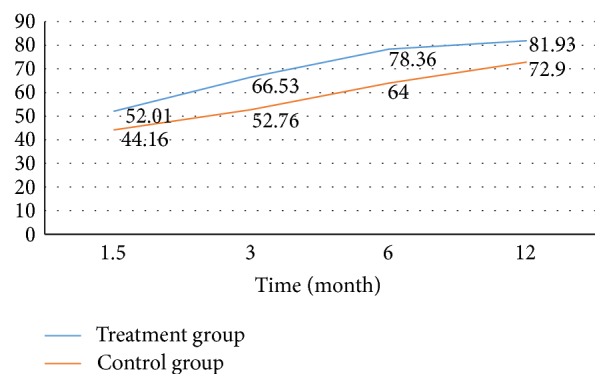
Intergroups changes on the Harris hip scores for 12 months.

The primary goal in the treatment of hip fracture is to bring the patients to the prefracture functional status. Postoperative functional status of the patients was evaluated with Harris hip score and the Merle d'Aubigné score. Harris hip score is commonly used by orthopedic surgeons to determine pain status of the patients, to assess normal daily activities of the patients, and to provide information about hip joint range of motion [32, 41]. Merle d'Aubigné scoring is done by evaluating pain, walking ability, and joint range of motion [32, 42]. Knobe et al. used Merle d'Aubigné and Harris hip score for comparing functional results of unstable intertrochanteric hip fractures [32]. In our study, 86 patients whose follow-up was completed at the end of postoperative 1st year were functionally assessed with Harris hip score and the Merle d'Aubigné scale at the 6th week, 3rd month, 6th month, and 12th month (Tables 2 and 3). While the average HSS was 81,93 in treatment group at the end of postoperative 1st year, it was found to be 72.9 in control group. While 83.4% (40/48) excellent and very good results were achieved in treatment group, 57.9% (22/38) excellent and very good results were obtained in control group. Similar results were also seen in the Merle d'Aubigné score. While 66.7% (32/48) excellent and good results were obtained in treatment group, 26.4% (10/38) excellent and good results were obtained in control group (Tables 2 and 3). In terms of functional results, Harris hip score and Merle d'Aubigné score showed parallelism and remained consistently higher in the zoledronic acid group from the 6th week (Figures 1 and 2). Amanat et al., in a study conducted on rats with diaphyseal femur fractures, showed that zoledronic acid increased the amount of callus, the mineral content, and durability 6 weeks after the fracture, and particularly the concentration was condensed in the fracture line [43]. Considering that osteosynthesis has been implemented to all of our patients, higher functional scores from the 6th week in the group with zoledronic acid may have been associated with this condition (Figures 1 and 2). But we believe that further work is needed on this issue.

The mortality rate in the intertrochanteric fractures varies between 15 and 30% [4, 30, 44] and most of them occur within the first year [30, 45–48]. Moran et al. found that mortality rates are 9% within the first 1 month, 19% within the first 3 months, and 30% within the first year [45]. Davidson et al. found that the mortality rate is 26% within one year after the hip fracture at a hospital in New Zealand and 15–25% in other studies [49, 50] and stated that the highest risk period was the first 6 months. Also mortality rates of the patients at the end of 8–12 months are equal to those who have not had any hip fractures and therefore it is possible to consider the patients being recovered at the end of one year [51, 52]. In our study, mortality rate of 114 patients with the mean follow-up time of 9.54 months because of the deaths has been found as 24.5% (28/114). The patients' time of death has been determined to be 57.1% (16/28) in the first 3 months and 92.9% (26/28) in the first 6 months and has been found to be consistent with the literature. There are a number of studies showing that zoledronic acid decreases the mortality rate after hip fractures [14, 18, 53, 54]. In our study, while mortality rate in treatment group was 14.3% (8/56), mortality rate in the other group was determined to be 34.5% (20/58). While the mean follow-up time was 10,6 months in treatment group, it was calculated as 8.4 months in control group. It was remarkable that deaths in both groups occurred within the first 3 months and we believe that this situation should be investigated.

Low number of patients despite having been determined by power analysis and not having used any radiological scoring, inability to assess joining time, the groups being nonhomogeneous because of the dying patients, and absence of comparison group of treatment with the other antiosteoporosis treatment constitute insufficient points of the study.

## 5. Conclusion

The use of zoledronic acid after surgical treatment of intertrochanteric femoral fractures in osteoporotic over 65-year-old patients is a safe treatment modality which helps to reduce mortality, improves functional outcomes, and has less side effects with single dose use per year.

## Figures and Tables

**Table 1:** Comparisons of the mean Merle d'Aubigné scores at each time point.

Merle d'Aubigné score	Treatment group (*N* = 48)	Control group (*N* = 38)	Total (*N* = 86)
6 weeks	7.96 ± 2.50	7.26 ± 3.09	7.65 ± 2.78
3 months	11.54 ± 3.03	9.37 ± 2.87	10.58 ± 3.13
6 months	13.63 ± 3.33	11.11 ± 3.18	12.51 ± 3.48
12 months	14.58 ± 3.08	12.68 ± 2.61	13.74 ± 3.02
*p* value	0.020	0.000	

**Table 2:** Comparisons of the mean Harris hip scores at each time point.

Harris hip score	Treatment group (*N* = 48)	Control group (*N* = 38)	Total (*N* = 86)
6 weeks	52.01 ± 16.92	44.16 ± 18.00	48.54 ± 17.74
3 months	66.52 ± 15.80	52.75 ± 17.52	60.44 ± 17.86
6 months	78.36 ± 14.86	64.00 ± 17.58	72.01 ± 17.55
12 months	81.93 ± 15.04	72.90 ± 14.55	77.94 ± 15.42
*p* value	0.025	0.000	

**Table 3:** Intergroup and intragroup paired *t*-test for changes in *T* scores which shows that zoledronic acid provides significant increase at the bone mineral density.

	Treatment group (*N* = 48)	Control group (*N* = 38)	*p* value
Pre-op DEXA	−2.70 ± 1.01	−2.75 ± 1.19	0.812
Post-op DEXA	−2.17 ± 1.10	−2.39 ± 1.16	0.354
*p* value	<0.001	0.059	

**Table 4:** Analysis of mortality rates between the groups with chi-square^*∗*^ tests which shows that zoledronic acid provides significant decrease at the mortality rates.

	Treatment group *N* (%)	Control group *N* (%)	Total *N* (%)
Alive	48 (85.7)	38 (65.5)	86 (75.4)
Dead	8 (14.3)	20 (35.5)	28 (24.6)
Total	56 (100.0)	58 (100.0)	114 (100.0)

^*∗*^Pearson chi-square *p* = 0.012.
